# Paleoclimate and bubonic plague: a forewarning of future risk?

**DOI:** 10.1186/1741-7007-8-108

**Published:** 2010-08-27

**Authors:** Anthony J McMichael

**Affiliations:** 1 National Centre for Epidemiology and Population Health, Building 62, Mills Road, The Australian National University, Canberra, ACT 0200, Australia

## Abstract

Pandemics of bubonic plague have occurred in Eurasia since the sixth century ad. Climatic variations in Central Asia affect the population size and activity of the plague bacterium's reservoir rodent species, influencing the probability of human infection. Using innovative time-series analysis of surrogate climate records spanning 1,500 years, a study in *BMC Biology *concludes that climatic fluctuations may have influenced these pandemics. This has potential implications for health risks from future climate change.

See research article http://www.biomedcentral.com/1741-7007/8/112

## Commentary

Today's diverse populations within the vast Eurasian continent, whether east, west, central or south, retain a horror of 'the plague' - as dreadful an agent of gruesome death as Ebola virus and yellow fever. Over the past two millennia, several pandemics of bubonic plague, caused by the flea-borne bacterium *Yersinia pestis*, have occurred within Eurasia, spreading quickly and often then lingering. Using a stepped approach to a set of long historical time-series data, including climatic, pandemic, epidemic and social-political variables, a study by Kausrud and colleagues [1] published in *BMC Biology *concludes that naturally occurring climatic fluctuations, acting through their environmental, ecological and political impacts, may have influenced the human pandemic outbreaks.

Descriptions and theories about the occurrence of bubonic plague, particularly the Black Death (estimated to have killed one-third to one-half of Western Europe's population), have engrossed many medical historians. In particular, the two great, recognized historical pandemics of bubonic plague have spawned various controversies.

The first was the Justinian Plague of 542 ad, which devastated Constantinople (by then the seat of the embattled Roman Empire). That great outbreak spread to engulf the greater Eastern Mediterranean region during the later sixth and seventh centuries. Second, in the 14th century, was the pandemic extending from China, through Central Asia, and eventually reaching Europe (the Black Death). Both pandemics occurred when great and complex political structures were becoming vulnerable. Did the Justinian Plague contribute to the terminal weakening of the eastern Roman Empire [2]? Did the Black Death hasten the collapse of Europe's feudal system, and the advent of liberalizing moves towards mercantilism, literacy and the Renaissance [3]? (And was the rise and fall of the Mongol-controlled Yuan Dynasty in China, from the mid-12th to mid-13th centuries, influenced by flickering pre-pandemic plague epidemics in China during that Medieval Warm period?)

## The central research question

Kausrud and colleagues examine whether changes in climatic conditions over the past 15 centuries have potentiated these main excursions of the plague bacterium from its Central Asian, ground-burrowing, rodent host populations into human populations. They have used impressively extensive and detailed historical data for Central Asia (Kazakh region). Even so, when delving back over so many centuries, surrogate measures of paleoclimatic conditions are necessary - in this case, tree-rings, glaciers and stalagmites. The authors have also had to make simplifying assumptions about the determinants of (and, hence, the proxy index for) the changeable level of sylvatic (rodent) plague activity over time.

This research task is complex, and some readers will find the analytic methods and the inferences drawn as challenging as they are innovative. The initial fine-tuning of the model to estimate (from climatic conditions and, hence, vegetation cover) the changeable level of sylvatic plague activity within rodent populations over past centuries was achieved by the empirical comparison of 10,000 (yes, 10,000) computer-generated model variants tested against 20th century Kazakh data. Each variant comprised a slightly differing combination of the several proxy measures of climatic conditions - temperature, rainfall, and monsoonal shifts - and their own many possible representations.

This study is timely, as the world community is now increasingly attentive to the likely impacts of human-induced climate change on the occurrence of infectious disease [4,5]. For many infectious agents, the probability of transmission is influenced variously by temperature, rainfall, humidity and wind patterns. The relationships between these factors are sometimes complex, and estimates of the impact of climate change on infectious disease have been contested [6]. Overall, though, it is certain that changes in climatic conditions will reset the boundaries, spatial and seasonal, on the transmission of many infectious diseases.

Salmonella food poisoning (diarrheal disease) occurs most often in summer, reflecting the faster proliferation of bacteria at higher temperatures. Cholera outbreaks occur more readily when coastal waters warm or when heavy rains cause flooding. The malarial parasite matures faster within the mosquito at warmer temperatures, and mosquito breeding, biting and survival are sensitive to temperature, surface water and humidity [4]. The northern limits of schistosomiasis transmission in China are set by the mid-winter 'freezing zone' - at which temperature the pathogen's intermediate host, the water snail, cannot survive [7]. In warmer waters, development of the schistosomiasis parasite within the snail can only occur above 15.4°C.

For bubonic plague it is entirely possible that the natural reservoir populations of rodents would have been affected by regional climate changes, including impacts on regional vegetation food sources. Such a relationship has been previously postulated [8]. In the current work, Kausrud *et al. *[1] focus on the level of sylvatic plague activity in the multi-decadal period preceding each of the three major pandemic outbreaks of plague and the Manchurian epidemics of the early 20th century.

## Drawing causal inferences

**Figure 1:**
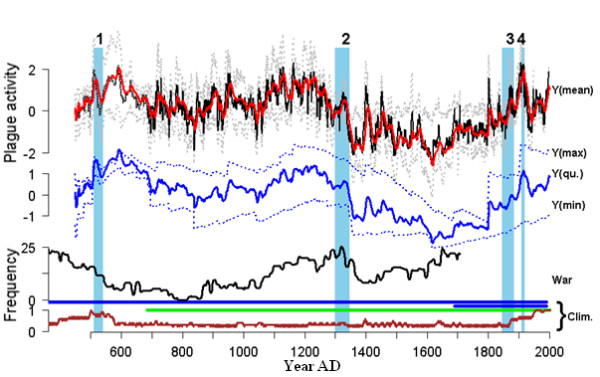
**Modeling the effects of climate on plague**. In the top plot, the solid black line represents plague activity in the central Asian rodent population (Y(mean)) over the past 1,500 years, as estimated from the authors' model of the effects of climate (including via observably correlated vegetation indices) on this natural reservoir (sylvatic) plague activity. The broken gray lines show 95% quantiles and the red line represents the multi-frequency (2 to 60 years) Gaussian moving average. The dark-blue plot represents the long-term (2 to 400 years) multi-frequency mean, with the maximum (upper broken line, Y(max)), minimum (lower broken line, Y(min)) and sum of minimum and maximum (solid line, Y(qu.)). The periods leading up to the Justinian Plague (1), the Black Death (2), the 19th-century pandemic (3) and the Manchurian epidemics (4) are shaded in pale blue. The third plot shows the index of conflict between Chinese and nomad societies (solid black line, War). Below this are shown the coverage of the climatic data used in the modeling: glacial series (blue), tree-ring index (green), and the decadal coverage in the monsoon proxy (brown). Taken from Figure 3d of Kausrud *et al. *[1].

The relationship is summarized in Figure 1. The figure indicates a rise in plague activity (seen best in the red graph) in the bacterium's natural homeland environment of Central Asia in the decades (often totaling a century or more) before spillover into human populations. Is that the critical influence - a prolonged multi-decadal increase in infected rodent numbers, as food sources expand and rodents and their fleas feast? And might the rapid decline in plague activity in the decade immediately preceding human spillover, evident for events 1, 2 and 4 in Figure 1, signify an acute reversal of fortune for rodent populations - disrupting colonies and displacing distressed animals, and thus facilitating human contact?

One cannot draw precise or certain inferences from these data. Nor is a test of statistical significance that compares many hundreds of model runs as informative as would be a test of some hypothesized, temporally specific relationship between plague activity and human spillover. Nevertheless, there are contemporary analogs for assorted climatic influences on the transmissibility of a vector-borne infection, including some with reservoir non-human hosts. Examples of the latter, each with major mammalian reservoirs upon which the 'vector' insects mostly feed, are Lyme disease in North America [9] and tick-borne encephalitis in Sweden [10]. Spillovers into humans in both cases appear to be influenced by climatic changes.

Whereas regional climate may influence local vegetation, rodent proliferation and infected flea numbers, long-distance transport of the pathogen typically requires human agency. Kausrud *et al. *[1] note that 'the two main periods of border expansion, migration and warfare by central Asian nomad pastoralists found in Chinese records, and known in European history from the Hun invasion of the 5^th ^century and the Mongol expansions of the 13^th^, are consistent with periods of high productivity in Central Asian grasslands having occurred prior to the great plague pandemics.' Indeed, this offers a clue to the apparent delay (Figure 1) between the high level of Central Asian plague activity in the 12th and 13th centuries and the outbreak of the Black Death in mid-14th-century Europe. The warmer, wetter, and hence grassier, steppes fueled the expansion of the horse-borne Mongol armies, who then extended their murderous reach westwards.

Meanwhile, the source of the Justinian Plague has long been disputed. Did it come from the east, or, as seems more likely, from Egypt and perhaps Ethiopia further down the Nile [2]? In this study the tree-ring time-series data (proxy for temperature) extend back only to the late seventh century. No clear answer is possible.

## Other challenges, present and future

There is another twist in the postulated relationship between climate and plague. The pandemics may themselves have affected the world's climate [11]. A prime determinant of the total human-generated emissions of greenhouse gases, at any one time, is human population size. The paleoclimatic record shows downward dips in atmospheric concentrations of carbon dioxide and methane during times of plague-related depopulation - when forest clearing would have slowed, animal husbandry receded and rice growing declined.

Twists aside, this study illustrates the usual epidemiological research challenge of discerning a signal against considerable background noise. The plague bacterium is a recent descendant of *Yersinia *ancestors such as *Y. enterocolitica *and *Y. pseudotuberculosis. *In parts of China, local pig and rodent populations infected by those two older pathogens display immunity to the 'plague' pathogen, *Y. pestis*. As humans too can be infected by these older forms, usually via the food chain, immunity to *Y. pestis *in human populations in (unhygienic) past times may have been quite high. That would add a further variable to an already complex mix; another potential time-related confounder to address. Meanwhile, innovative research initiatives - spanning bacterial DNA retrieval from human skeletons, analysis of inscriptions on gravestones, and evidence of fluctuations in building activity (engraved dates) - are enriching the microscopic and macroscopic archeology of plague research.

As Kausrud and colleagues [1] point out, though, today's immediate challenge is to become better informed about the risks that human-induced climate change poses via impacts on major infectious diseases. Their innovative study has drawn, enterprisingly, on a set of long-run time-series data. It suggests that this ancient dread disease, bubonic plague, may not yet have run its course. We are on notice.
